# Screening of Commercial Organic Solvent Nanofiltration Membranes for Purification of Plastic Waste Pyrolysis Liquids

**DOI:** 10.3390/membranes13090792

**Published:** 2023-09-12

**Authors:** Rick van Lin, Paulina A. Sosa Fernandez, Tymen Visser, Patrick de Wit

**Affiliations:** 1 Membrane Science and Technology Cluster, University of Twente, Drienerlolaan 5, 7522 NB Enschede, The Netherlands; r.vanlin@emi-twente.nl (R.v.L.);; 2 EMI Twente B.V., Drienerlolaan 5, 7522 NB Enschede, The Netherlands

**Keywords:** organic solvent nanofiltration (OSN), plastic pyrolysis, circularity

## Abstract

Increasing consumption rates of plastics, combined with the waste generated from their production, leads to several environmental problems. Presently, plastic recycling takes account of only about 10% of the plastic waste, which is achieved mainly through mechanical recycling. Chemical recycling methods, such as pyrolysis, could significantly increase overall recycling rates and reduce the need for the production of fossil-based chemicals. Produced pyrolysis oil can be used for the production of benzene, toluene and xylene (BTX) through catalytic upgrading or for the production of alkanes if used directly. Separation of high-value components in pyrolysis oil derived from plastic waste through traditional separation methods can be energy intensive. Organic solvent nanofiltration has been recognised as an alternative with very low energy consumption, as separation is not based on a phase transition. This work focuses on the screening of several (semi-) commercially available membranes using a simplified model mixture of pyrolysis oil obtained from plastics. Based on membrane performance, a selection of membranes was used to treat a feedstock obtained from the direct pyrolysis of plastics. This work shows that currently, commercial OSN membranes have promising separation performance on model mixtures while showing insufficient and non-selective separation at very low flux for complex mixtures derived from the pyrolysis of plastics. This indicates that OSN is indeed a promising technology but that membranes should likely be tailored to this specific application.

## 1. Introduction

Plastics are one of the most versatile materials and their use has spread exponentially in the last decades. However, up to date, end-of-life management of plastic products has not been properly addressed. By the year 2015, humans had produced 8.3 billion tons of plastic, of which 6.3 billion tons had already become waste [1]. Plastic waste accounts for about 8–12% of the total municipal solid waste generated [2]. Since the 1950s, almost 50% of all plastic has accumulated in landfills or in natural environments, while only 9% has been adequately recycled [3]. In addition, since the production of over 90% of virgin plastics is based on fossil fuels, increasing plastic recycle rates is a fundamental part of the effort to minimize their exploitation and to reduce associated emissions [4].

There are several methods to recycle plastic, usually classified in four types: primary, secondary, tertiary and quaternary. In primary and secondary methods, the plastic is treated via mechanical methods and reused; in the tertiary method, waste plastic is chemically treated in processes that generate chemicals and fuels; and in the and quaternary methods, the plastic is incinerated for energy recovery [4]. Since mechanical recycling works best with separately collected plastic and fails to efficiently recycle mixed plastic waste [5], chemical (or tertiary) processes have high potential to be implemented as these methods typically accept these mixed plastic streams.

Chemical processes for plastic recycling can be classified in five categories [6]: depolymerization by reaction with certain agents to yield the starting monomers; gasification with oxygen and/or steam to produce synthesis gas; thermal decomposition of the polymers by heating in an inert atmosphere; catalytic cracking and reforming; and hydrogenation (the polymer is degraded by the combined effects of heat, hydrogen and in many cases catalysts). Each of these methods has advantages and restrictions, and the products obtained from them differ in quality and value, which makes the selection of a certain technology case-dependent. For example, for gasification a minimum supply of 400,000–500,000 tonnes of plastic per year is necessary to make the plant economically viable [6].

Among the chemical processes, pyrolysis is a promising option. Pyrolysis is a chemical recycling technology that can utilize a broad range of plastics and that has been recognized as an ideal approach to recover energy and obtain high-value products. Pyrolysis achieves the degradation of organic materials under the effect of heat and in an oxygen free environment. It requires temperatures between 400 and 700 °C, yielding up to 80 wt.% liquid yield. If process efficiency needs to be improved or if specific reactions are targeted, catalytic pyrolysis may be used. This typically yields a liquid with characteristics similar to traditional diesel fuel [5,7]. The obtained pyrolysis oil can be either upgraded to be used as a fuel, or further refined by extracting high value components such as benzene, toluene and xylene [8]. In any case, the pyrolysis oil requires purification before any intended application.

There are several established technologies which are commonly used to purify pyrolysis oil, including extraction, adsorption and distillation, the latter being the most widespread [9]. However, the high energy intensity of distillation and other thermal technologies is an incentive for the development of less energy-dependant methods, including column chromatography, membranes, electrosorption and extraction by ionic liquids [10]. Among these, the use of membrane technology is appealing due to its low energy consumption, flexibility, operational simplicity and scalability [10].

Despite its advantages, the application of membrane solutions for liquid treatments has been mainly focused to aqueous applications. Solvent-resistant nanofiltration (SRNF) or organic solvent nanofiltration (OSN) has shown promising results incorporating membrane technology in the presence of organic solvents [11]. Since OSN does not rely on phase changes, the energy consumption can be less than 5% of the energy spent for distillation [12]. In addition, this process generally has no need of using extra additives, as is the case in other separation technologies. Lastly, separation of heat-sensitive components can be recovered with low chances of thermal degradation [13,14]. Given all these advantages, OSN has proven to be an ecologically and economically sound alternative to thermal separation processes in fields such as the pharmaceutical, fine-chemical, petrochemical and biochemical industries [15].

In this work, we demonstrate the use of commercially available OSN membranes for the purification of pyrolysis oil derived from plastics. To the authors’ best knowledge, this is the first time that plastic-derived pyrolysis oil is attempted to be purified by means of a membrane system. The experimental work included an initial screening of commercial membranes, tested with a simplified model mixture of the pyrolysis oil, and further testing of the most promising membranes with real pyrolysis oil derived from plastic pyrolysis. The results obtained included the fluxes of different components as well as fouling analysis of one of the membranes.

## 2. Materials and Methods

### 2.1. Membranes

Membrane samples were obtained from various suppliers; their details are given in Table 1. In order to screen membranes, they were pre-treated according the specific manufacturer instructions. For flat sheet membranes, they were cut to the desired size and placed in the DIN A7 sized pressure housing. A 0.7 mm spacer was installed to promote turbulence during flat sheet testing.

### 2.2. Chemicals and Model Mixtures

n-Octane (99+%), n-hexadecane (99%) and 1-hexene (97%) were received from Acros (Belgium). Cumene, 1-methylnaphthalene and toluene (99.5%) were received from Sigma Aldrich. N-heptane was received from Alfa Aesar. Pyrolysis liquids were kindly supplied by Urbaser, Spain. All chemicals were used as received. Table 2 shows the composition of the model mixture used in this study, whereas the chemical composition of the real pyrolysis mixture is given in Table 3.

### 2.3. Experimental Setup and Procedure

The crossflow setup that was used consisted of a 5 L feed vessel from which a dedicated HPLC pump pressurized a circulation loop between 20 and 30 bar by means of a backpressure valve. The feed and retentate are thus both pressurized. The crossflow velocity over a flat membrane active area of 2.8 cm^2^ was generated and controlled by a separate circulation pump. The flat sheet membranes are mounted, including a 0.7 mm spacer, in a DIN A7 sized cell. Ceramic modules were installed in a tubular SS-316 housing, using O-rings for sealing. The setup is pressurized to 33 bar and at least 50 mL is permeated, and discarded, per membrane sample. After this, the setup is operated in full recycle mode where both retentate and permeate are fed back to the feed vessel for 2 h, after which steady state is assumed. Per membrane type, two coupons are measured at 20, 25 and 30 bar transmembrane pressure. Permeate flow is measured volumetrically by collecting permeate using a measurement cylinder and recording the time required to reach a given volume. To minimize measurement errors, at least 6 readings are taken over a 10–15 min interval and the permeate flow is calculated using linear regression of these measurement points. All membranes are screened at a feed crossflow velocity above 0.3 ms^−1^. The temperature of the feed solution is controlled at 30 °C. The potential of each membrane was screened using a model mixture in order to assess membrane suitability. Suitable membranes were further characterized using a more complicated (model) mixture or using real (pyrolysis) liquids.

The flux through the membrane (*J*, in Lm^−2^ h^−1^) was calculated using the following equation:

(1) J=V÷(A·t)

where *V* is the permeate volume (L), *A* the membrane area (m^2^) and *t* is the permeation time (h). The permeance coefficient, *P* (Lm^−2^ h^−1^ bar^−1^), was calculated from the slope of the flux versus trans membrane pressure (TMP) graph:

(2) $$P=\frac{J}{\Delta P}$$

### 2.4. Analysis

Samples are taken from the feed and permeate side and are analyzed using GC-MS (GC 7890A MS 5975C—Agilent Technologies, Santa Clara, CA, USA) using a Agilent HO5-MS, HP19091S-433 capillary column with helium (2 mL/min) as carrier gas. Initial column temperature was 45 °C, ramping 3 °C/min to an end column temperature of 280 °C. Relative concentration of the components is derived from peak areas and the total peak area. Retention is calculated based on the concentration difference in feed and permeate using the following equation:

(3) $$R=1-(C_{p}÷C_{f})$$

### 2.5. Fouling

Two fouling-oriented experiments were performed. The first experiment consisted of permeating through one of the evaluated membranes in a full recycle mode and logging the permeate flow over a period of 300 h using a Bronkhorst miniCORI-FLOW M13 flowmeter. The reduction in permeance was measured in a different experiment in which pyrolysis oil was diluted with different portions of clean n-hexane. The fouled membrane from the first experiment was analyzed by means of Fourier-transform infrared spectroscopy (FT-IR) and thermal gravimetric analysis (TGA). FTIR spectra were measured with a Bruker Alpha. TGA measurements were performed on a Perkin Elmer TGA 4000 under nitrogen atmosphere at a linear heating rate of 20 °C/min. Samples (10 mg) were heated over a 30– 900 °C temperature range.

## 3. Results

In order to facilitate rapid screening of membrane separation performance, initial experiments were carried out using a simplified model mixture (Mixture A, see Table 2) consisting of C6–C8 alkanes and alkenes, in addition to toluene and n-hexadecane. Although the usage of model mixtures is debated in the OSN field [16,17], a first assessment can be useful in order to evaluate the membranes initial permeability and retention. Selection of a suitable model mixture remained challenging; ultimately, these mixtures were selected based on their ability to be easily differentiated on GC-spectra to include a mixture of similar size alkane-alkene-aromatic components and one larger alkane. For mixture B, two additional impurities were added to see the effect to of small trace components on the separability of the mixture.

### 3.1. Model Mixtures

#### 3.1.1. Flux

**Figure 1:**
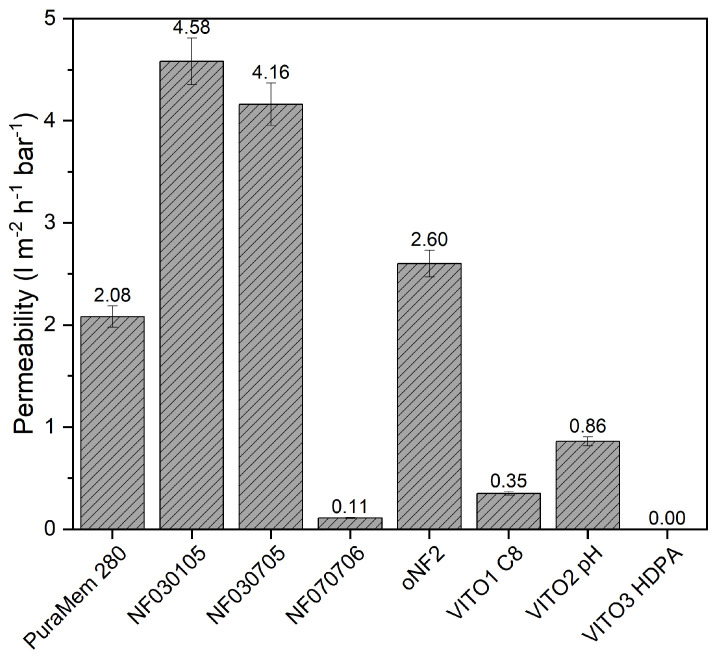
Permeance of commercial membranes using model mixture ‘A’ at (30 °C). Error bars represent an indication of the measurement error based on pressure gauges and flow measurement method.

The measured permeances of the commercial membranes tested on feed of Mixture A are shown in Figure 1. It can be seen that the permeances of the membranes ranged between 0.2 and almost 5 Lm^−2^ h^−1^ bar ^−1^, which is in good agreement with the values specified by the membrane manufacturers. The Vito HDPA membrane had the lowest permeance (too low for reliable retention measurements), whereas the NF030105 from SolSep showed the highest one. Several studies show single-solvent measurements with different types of solutes at similar permeabilites for Puramem 280, oNF2 [18,19,20] and VITO [16] membranes.

#### 3.1.2. Retention

**Figure 2:**
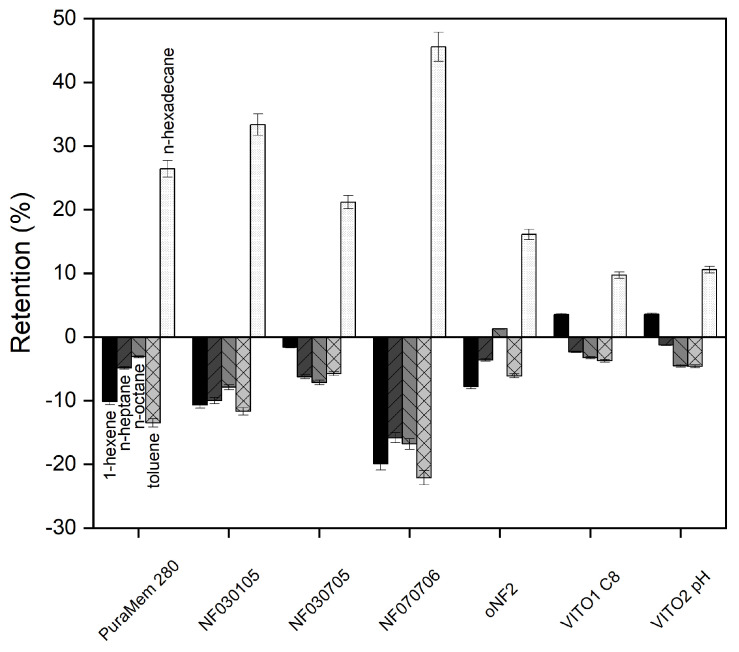
Retention of commercial membranes using model mixture ‘A’ at (30 °C). Error bars represent an indication of the measurement error, taking into account the estimated accuracy of the GC method.

The retentions of the compounds present in the model mixture A (n-octane, n-hexadecane, 1-hexene, toluene, n-heptane) are presented in Figure 2. It shows that almost all membranes presented a relatively good retention for aromatic or smaller alkene components. Yet, there is difference in degree of retention of the various compounds, allowing partial fractionation of the model compounds. For example, the Borsig oNF2 membrane shows a positive retention of n-hexadecane and octane, whereas it shows a negative retention for 1-hexene and toluene. This indicates the permeate will be enriched in aromatics and smaller alkenes/alkanes as compared to the feed mixture.

Based on the measured permeances, retentions and the availability at industrially relevant scales, the Evonik Puramem 280 and VITO C8 membranes were selected for further characterization using a more complex model mixture (Mixture B) and using various operating conditions. The NF070706 and VITO HDPA were discarded due to their low permeability with the model mixture, the oNF2, due to its retention. Although the NF030105 and NF030705 showed acceptable permeability and retention, they were not included due to their availability.

#### 3.1.3. Effect of Crossflow Velocity

Experiments were performed using Mixture B to determine the effect of adding two more components. By varying the crossflow velocity, it is possible to evaluate the effect of concentration polarization boundary layer size and the effect on retention. Crossflow velocities differ per membrane type as a result of different geometries. Puramem 280 is a flat sheet, and so it was mounted, including a feed spacer to promote turbulence. The Vito C8 membrane was mounted in a stainless steel housing and was operated inside-out. Both experiments were performed at 30 °C and 30 bar.

**Figure 3:**
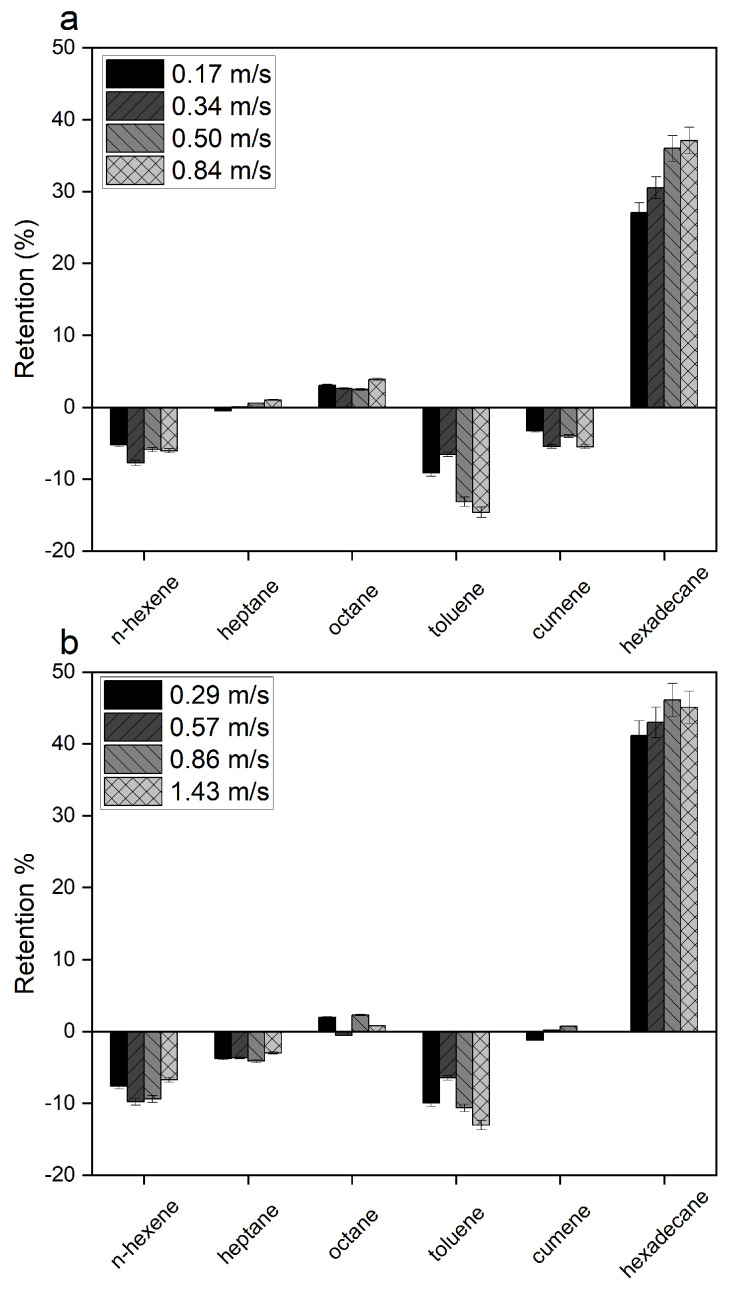
Effect of crossflow velocity on the retention of “Model Mixture B” at (30 °C) for (**a**) Puramem 280 and (**b**) Vito C8. Error bars represent the accuracy of the GC method.

The results of this test are summarized in Figure 3. This figure shows that the positive retention of n-hexadecane increases at higher crossflow velocity conditions while showing a decreasing retention for toluene. This results in a more optimal separation between higher alkanes and the aromatic compounds. It is hypothesized that this effect results from a smaller concentration polarization boundary layer due to higher turbulence near the membrane surface [21].

### 3.2. Experiments with Real Pyrolyis Oil

To assess the performance of membranes using real mixtures, experiments were carried out using a pyrolysis oil mixture originating from a LDPE/PP/PS blend at a ratio of 30/60/10, respectively. Table 3 shows the composition of this pyrolysis liquid analyzed by GC-MS.

Experiments were performed in full recycle mode at 30 bar operating pressure and 30 °C. The crossflow velocity is maintained at 0.3 ms^−1^ (Re = 3640) and 0.5 ms^−1^ (Re = 14,875 for the Evonik Puramem 280 and Vito C8, respectively.

#### 3.2.1. Flux and Retention

**Figure 4:**
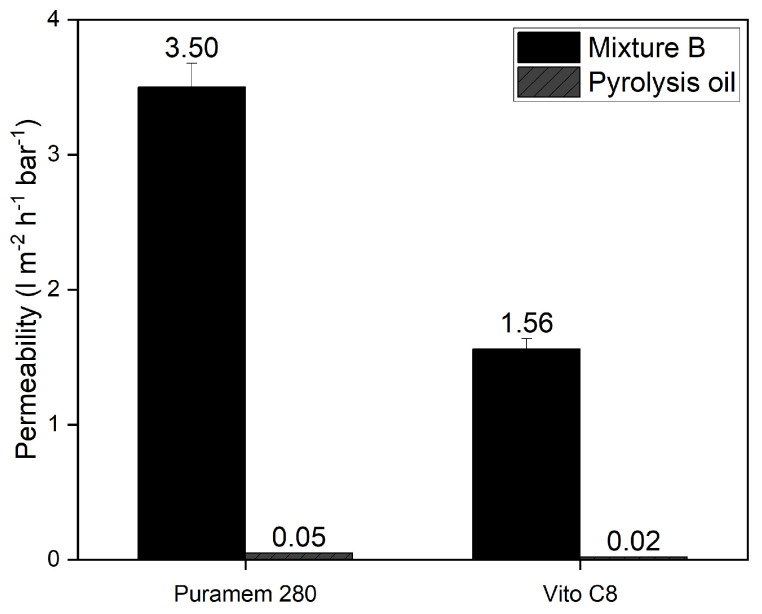
Difference between model and real mixture (30 °C). Error bars are based on the accuracy of the pressure gauges and flow measurement method.

Figure 4 compares the permeability when filtering the pyrolysis oil mixture against that obtained using Mixture B. As can be observed, the permeability is reduced by approximately 99% when using a real pyrolysis mixture. The lower permeability is most likely a result of a combination of higher osmotic pressure and (irreversible) fouling. In addition, it should be noted that organic solvent mixtures stray far from ideality [22]. Small changes in composition might have a large effect on the activity of components, thus resulting in different component flux.

Table 4 shows the retention of Evonik Puramem 280 and Vito C8 membranes. Both membranes show no clear selectivity to specific components. Slight retention of larger components such as 1,3,5-trimethylcyclohexane, 3,3,5-trimethylcyclohexane and 2,4-dimethyl-1-heptene is observed. The pyrolysis oil is not effectively separated using these membranes as there is no clear affinity for a single type of component, making separation difficult and non-selective. Also, the degree of error in these values is relatively large, originating from the low absolute concentrations of the components in the mixture and the used GC method.

#### 3.2.2. Fouling

**Figure 5:**
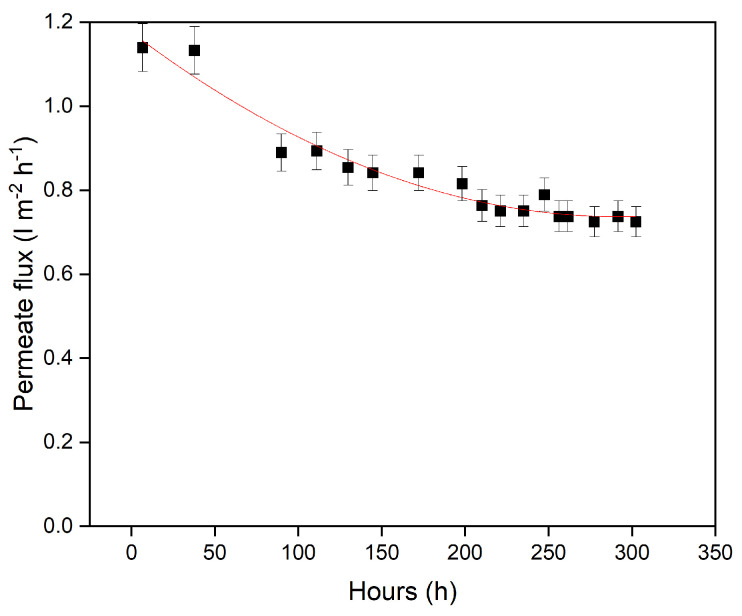
Permeance of Puramem 280 during long duration full recycle testing (30 °C, 30 bar). The red line is to guide the eye. Error bars are based on the accuracy of the pressure gauges and flow measurement method.

Significant fouling was observed during the membrane characterization experiments. Thus, a fouling experiment was performed using Evonik Puramem 280 membrane, where over a period of 300 h the permeate flow was logged, as shown in Figure 5.

**Figure 6:**
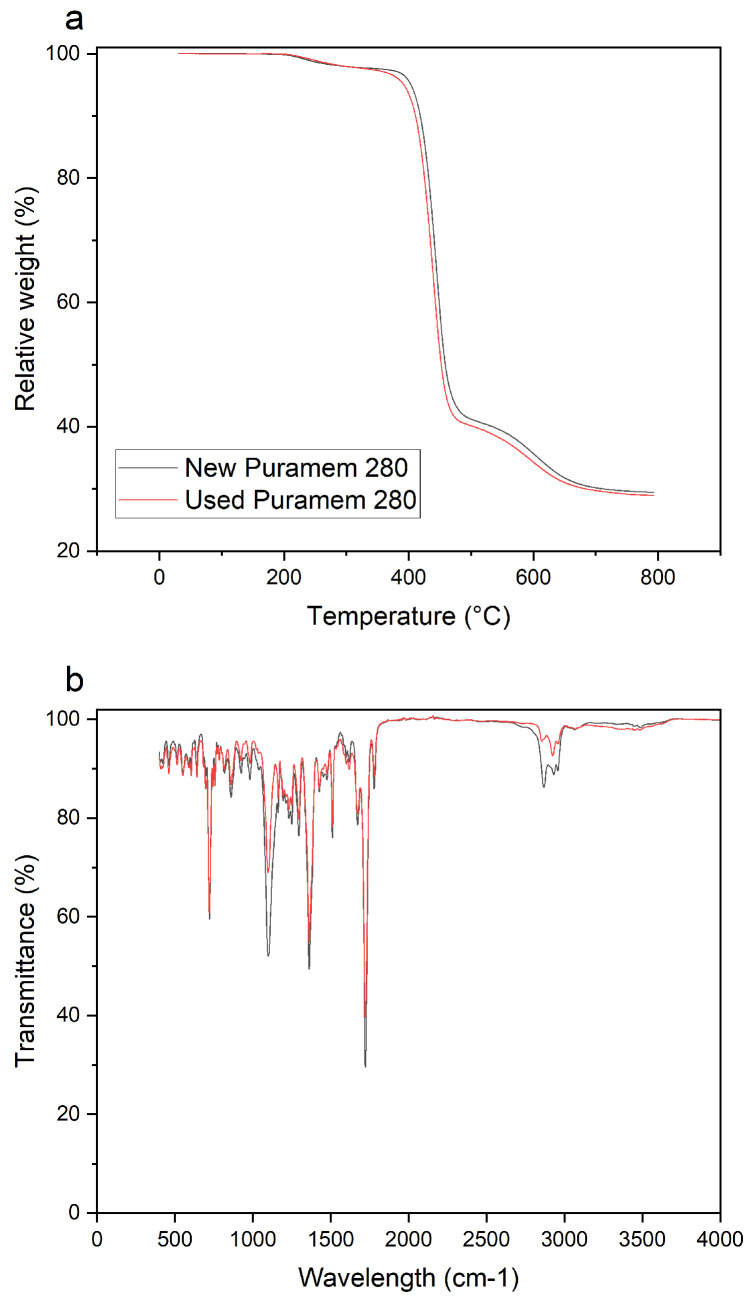
FTIR (**b**) and TGA (**a**) analysis of fouled and new Puramem 280.

After this run, a membrane autopsy was performed. Figure 6 compares FT-IR and TGA results of a new and fouled Puramem 280 flat sheet membrane, indicating no clear differences in surface chemistry. This indicates that surface modifications did not occur.

To investigate the reduction in permeance, filtration experiments containing various degrees of diluted pyrolysis oil were performed. By adding pyrolysis oil to the clean n-hexane it was possible to determine that if the reduction of permeance occurred gradually-suggesting fouling of the membrane-or almost instantaneous, which would suggest a sorption-like mechanism of one of the components in the mixture.

**Figure 7:**
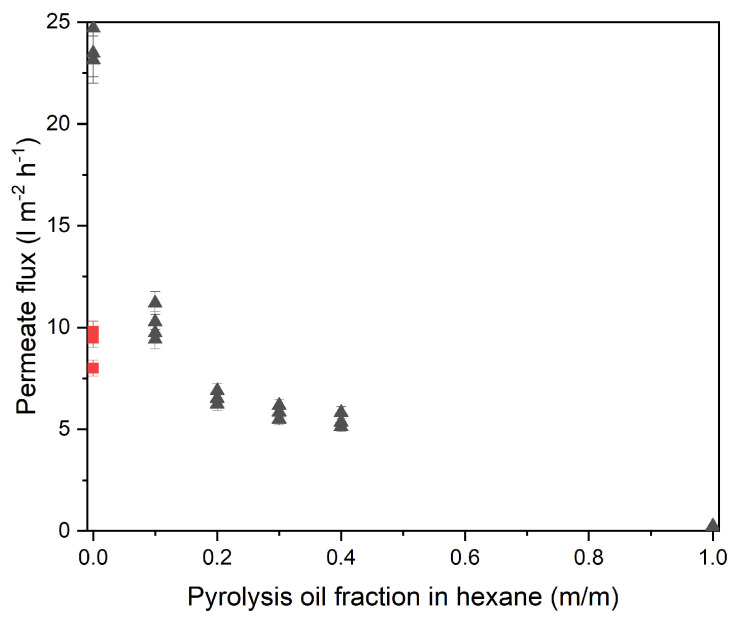
Permeance decline of Puramem 280 as function of pyrolysis oil fraction in n-hexane (30 °C, 30 bar). Error bars are based on the accuracy of the pressure gauges and flow measurement method. Black triangles indicate the measured permeate flux at various pyrolysis oil fractions. Red squares represent a clean n-hexane measurement after the pyrolysis oil fraction is increased and decreased. Three samples measured at each pyrolysis oil fraction.

Permeance experiments were started using clean n-hexane, and step-wise the pyrolysis oil fraction was increased. The membrane flux was measured at steady state before pyrolysis oil content was increased. Figure 7 shows how a small amount of pyrolysis oil results in a large reduction of permeate flux. This suggests that one or more components in the feed affects the membranes permeability and rules out any fouling or viscosity effects. Upon increasing the concentration of the pyrolysis oil further, the flux continues to decline until it becomes almost zero. After flushing with n-hexane followed by a n-hexane permeance experiment, the membrane does not recover (indicated by the red square). This suggests that one or multiple components in the mixture remain present in the membrane structure, potentially affecting the separation mechanism of the membrane.

## 4. Discussion and Conclusions

The separation of a complex pyrolysis oil mixture derived from the pyrolysis of plastics was tested with commercially available OSN membranes. Preliminary tests with a model mixture showed separation of higher molecular weight alkanes from lower molecular weight components was achieved while maintaining acceptable flux when using the Evonik Puramem 280 and Vito C8 membranes. Then, when the same membranes were tested with real pyrolysis oil, insufficient and non-selective separation at very low flux was observed for complex mixture derived from the pyrolysis of plastics. Irreversible fouling was observed, which was explained by strong sorption of one or multiple components in the mixture. For the complex pyrolysis oil mixture studied here, no commercially available membranes are suitable to perform the desired separation.

In our opinion, taking a holistic approach and tuning the pyrolysis process more to the suitability of the OSN membranes, e.g., adding an integrated catalytic step, could potentially resolve this. Additionally, further optimizing the process conditions such as dilution of the pyrolysis mixture, or increasing the temperature, could further aid the separation performance. Another option would be to develop OSN membranes specifically for pyrolysis oils, including some fouling resistant properties. A final alternative route could be to combine OSN with other separation methods, like fractionated condensation or distillation, to decrease the energy requirements for separation.

## Figures and Tables

**Table 1:** Properties of the (pseudo)commercial membranes used in this study. Both thin film composites (TFC) and ceramic tubular modules were tested.

Supplier	Type	Batchno.	Membrane Architecture	Material	
				Support	Selective Layer
Evonik	Puramem 280	M257/1	TFC	Polyimid	Proprietary
SolSep	NF030105	1808S	TFC	Proprietary	Proprietary
SolSep	NF030705	1905S	TFC	Proprietary	Proprietary
SolSep	NF070706	1810S	TFC	Proprietary	Proprietary
Borsig	oNF-2	2844	TFC	Proprietary	Proprietary
VITO	C8	n/a	Ceramic, tubular	TiO_2_	Proprietary Grignard-grafted
VITO	Ph	n/a	Ceramic, tubular	TiO_2_	Proprietary Grignard-grafted
VITO	HDPA	n/a	Ceramic, tubular	TiO_2_	Proprietary Grignard-grafted

**Table 2:** Composition of model mixtures used in this study.

Compound	Mix A	Mix B	MW (g mol^−1^)
n-Octane	20.0%	19.5%	114.2
n-Hexadecane	20.0%	9.7%	226.4
1-Hexene	10.0%	24.3%	84.2
Toluene	25.0%	15.4%	92.1
n-Heptane	25.0%	20.6%	100.2
Cumene	0.0%	10.2%	120.2
1-Methylnaphthalene	0.0%	0.1%	140.2

**Table 3:** Composition of real pyrolysis liquid. Compounds below 0.5 wt% are not reported.

Compound	wt%	MW (g mol^−1^)
2,4-Dimethyl-1-heptene	22.4%	126.2
Pentane	14.8%	72.2
2-Hexene, 5-methyl	11.2%	96.2
Toluene	10.2%	92.1
Styrene	5.6%	104.2
2-Pentene, 3-methyl-	5.0%	84.2
Benzene, 1-methylethyl	4.8%	120.2
Octane	4.6%	114.2
Heptane, 4-methyl-	4.5%	114.2
Ethylbenzene	3.3%	106.2
1-Propene, 2-methyl-	2.6%	56.1
Heptane	2.4%	100.2
Cyclohexane, 3,3,5-trimethyl	2.1%	124.2
1,3-Pentadiene, 2-methyl	2.0%	82.1
Methylstyrene	1.6%	118.2
2-Pentene, 4-methyl-	1.5%	84.2
Cyclohexane, 1,3,5-trimethyl-	0.8%	124.2
Pentane, 2-methyl-	0.5%	86.2
1-Pentene, 2-methyl-	0.5%	84.2
1-Heptene	0.5%	98.2

**Table 4:** Retention of Evonik Puramem 280 and Vito C8 using pyrolysis oil at 30 °C.

	280	C8
Compound	Retention	
Cyclohexane, 1,3,5-trimethyl-	+14.5%	+14.1%
Cyclohexane, 3,3,5-trimethyl	+10.9%	+12.0%
2,4-Dimethyl-1-heptene	+7.7%	+7.9%
Octane	+3.8%	+3.6%
2-Pentene, 3-methyl-	+3.5%	+0.0%
Heptane, 4-methyl-	+1.9%	+2.9%
Benzene, 1-methylethyl	+1.4%	+2.5%
Heptane	+0.2%	−0.9%
1,3-Pentadiene, 2-methyl	−1.9%	+0.5%
Ethylbenzene	−2.1%	−3.5%
Methylstyrene	−2.3%	−0.2%
2-Hexene, 5-methyl	−2.9%	−3.9%
2-Pentene, 4-methyl-	−4.4%	−7.4%
Pentane	−4.7%	−3.3%
Toluene	−4.7%	−5.8%
Styrene	−5.3%	−5.0%
1-Pentene, 2-methyl-	−6.4%	−6.7%
1-Propene, 2-methyl-	−9.2%	+3.3%
1-Heptene	−9.8%	−12.4%
Pentane, 2-methyl-	−13.1%	−15.1%

## Data Availability

The data presented in this study are available on request from the corresponding author. The data are not publicly available.
